# Outcomes and treatment failure after open or robotic ureteral reconstruction for iatrogenic injuries

**DOI:** 10.1002/bco2.267

**Published:** 2023-07-04

**Authors:** Marta Rossanese, Gianluca Giannarini, Riccardo Scalia, Luciano Macchione, Alessandro Crestani, Alchiede Simonato, Vincenzo Ficarra

**Affiliations:** ^1^ Gaetano Barresi Department of Human and Paediatric Pathology, Section of Urology University of Messina Messina Italy; ^2^ Urology Unit Santa Maria della Misericordia University Hospital Udine Italy; ^3^ Department of Surgical Oncological and Stomatological Sciences, Section of Urology University of Palermo Palermo Italy; ^4^ Oncological Urology Unit Veneto Institute of Oncology IOV‐IRCCS Padua Italy

**Keywords:** complications, injury, reconstructive surgery, stricture, ureter

## Abstract

**Objectives:**

The aim of this study is to assess the aetiology, perioperative outcomes and treatment failure of contemporary patients undergoing open or robot‐assisted ureteral reconstruction for iatrogenic injuries.

**Patients and methods:**

We retrospectively analysed consecutive patients who underwent ureteral reconstruction for iatrogenic injuries at two academic centres 07/2013 and 06/2022. A variety of open or robot‐assisted procedures were performed, including uretero‐cystoneostomy, Boari bladder flap, uretero‐ureterostomy, ileal replacement and pyelo‐ureteroplasty. All procedures were performed by a single surgeon with extensive experience in open and robot‐assisted surgery and preference‐based management. Outcome measures were aetiology, estimated blood loss, length of stay, postoperative complications and treatment failure, defined as upper tract obstruction requiring permanent urinary drainage. We also planned a subgroup analysis comparing the outcomes between open and robot‐assisted procedures.

**Results:**

Fifty‐nine patients were included. Most injuries were consequent to endourological procedures (44%). The most frequently performed procedures were uretero‐cystoneostomy (49.2%) and Boari bladder flap (32.2%). Forty (67.8%) were open, and 19 (32.2%) were robot‐assisted procedures. Major postoperative complications were recorded in seven (11.9%) patients. After a median follow‐up of 42 months (interquartile range 12–24), treatment failure was observed in seven (11.9%) cases. Robot‐assisted versus open procedures were associated with decreased estimated blood loss (*p =* 0.01), length of stay (*p* < 0.001) and treatment failure (0/19 vs. 7/36, *p =* 0.04).

**Conclusion:**

In our series of iatrogenic ureteral lesions requiring reconstructive surgery, endourological procedures were the most frequent cause. Major postoperative complications were infrequent, and treatment failure rate was low. The robot‐assisted approach was associated with improved perioperative outcomes and lower failure rate compared with open.

## 1 INTRODUCTION

The incidence of iatrogenic ureteral injuries was historically estimated between 0.5% and 1% of all surgical procedures.^1^ However, in the last decades, an increasing number of these lesions have been observed in patients undergoing endourological interventions for the management of urinary stones or upper tract tumours.^2^, ^3^ More rarely, ureters can be damaged during other surgical or endoscopic urological procedures such as partial nephrectomy, retroperitoneal lymph node dissection, radical and simple prostatectomy and transurethral resection of the prostate or bladder tumours. Ureteral injuries can also occur after gynaecological, colorectal and vascular procedures.^4^, ^5^ Notably, the widespread use of abdominal and gynaecological laparoscopic surgery has apparently resulted in an increased risk of iatrogenic ureteral lesions.^6^ In the last years, spinal surgery has also been associated with these lesions.^7^

Iatrogenic ureteral injuries require prompt management in order to prevent severe sequelae, such as irreversible impairment of renal function and/or infections.^8^ Although upper tract drainage is frequently the first step in their management, surgical repair is required in cases with large defect/severe damage or after initial endoscopic treatment failure. Available studies of ureteral reconstruction are limited by the small samples and the heterogeneity in aetiology, with a mixing‐up of iatrogenic lesions with other conditions, such as ureteral tumours, stones, endometriosis, retroperitoneal fibrosis and others,^9^, ^10^ thus hampering strong recommendations.

Uretero‐cystoneostomy with or without bladder psoas hitch is the most frequent procedure performed in the management of pelvic ureteral iatrogenic injuries, which account for roughly 90% of all iatrogenic ureteral lesions.^8^, ^11^ Conversely, more complex reconstructive procedures, such as Boari bladder flap, uretero‐ureterostomy, pyelo‐ureteroplasty, buccal mucosa graft onlay, bowel replacement or renal auto‐transplantation, are considered in the management of the less frequent iatrogenic lesions involving the mid‐ and upper‐ureter.^5^, ^12^, ^13^, ^14^

Although laparoscopic ureteral reconstruction techniques have been proposed as a minimally invasive alternative to open approach, pure laparoscopy remains challenging due to the limited degree of freedom and absence of steadiness.^15^ To overcome these issues, some authors have proposed uretero‐cystoneostomy with or without bladder psoas hitch and/or Boari bladder flap using a robotic platform.^2^, ^9^, ^16^, ^17^

Whereas several series confirmed the efficacy of uretero‐cystoneostomy with or without bladder psoas hitch in the management of pelvic ureteral iatrogenic injuries, only few studies analysed the outcomes of reconstructive surgery for mid‐ or upper‐ureter lesions.^3^, ^8^, ^9^, ^10^, ^18^ Moreover, no study has ever compared perioperative outcomes and failure rate of open versus robotic ureteral reconstruction for iatrogenic injuries.

The primary objective of the present study was to assess aetiology, perioperative outcomes and treatment failure rate in a contemporary series of consecutive patients undergoing open or robot‐assisted ureteral reconstruction for iatrogenic ureteral injuries. As a secondary objective, we compared perioperative outcomes of open versus robot‐assisted procedures.

## 2 PATIENTS AND METHODS

We retrospectively analysed the clinical records of consecutive patients who underwent ureteral reconstruction for iatrogenic injuries 07/2013 to 04/2017 at Santa Maria della Misericordia University Hospital, Udine, Italy, and 05/2017 to 06/2022 at Gaetano Barresi Department of Human and Paediatric Pathology, University of Messina, Italy. Immediate reconstruction was considered in cases with intraoperative detection of large defect or severe damage. Conversely, patients who underwent delayed reconstruction had symptoms (fever, abdominal pain) and/or evidence of ureteral obstruction or leakage on standard imaging. All patients provided written informed consent for data collection and publication.

Patients receiving a conservative management (retrograde ureteral stent, percutaneous nephrostomy tube and/or endoscopic realignment) with no subsequent ureteric reconstruction, experiencing ureteral injuries due to blunt or penetrating trauma and undergoing ureteral reconstruction for urothelial tumours, stones, endometriosis, uretero‐ileal anastomotic strictures following radical cystectomy and ileal urinary diversion and other noniatrogenic conditions, or with previous radiation therapy to abdomen/pelvis were excluded.

From an anatomical standpoint, the ureter was divided into an abdominal tract (from the ureteropelvic junction to the crossing with iliac vessels) and a pelvic tract (from the crossing with iliac vessels to the orifice in the bladder). The abdominal ureter was further divided into three segments based on their different blood supply: immediately distal to the ureteropelvic junction, lumbar and crossing the iliac vessels.^3^

The following preoperative variables were assessed: age at diagnosis, gender, body mass index, Charlson comorbidity index, serum creatinine, estimated glomerular filtration rate (eGFR), cause of injury, timing of reconstruction, involved ureteral side and segment and type of upper tract drainage placed (retrograde ureteral stent or percutaneous nephrostomy tube). Preoperative imaging evaluation was based on abdominal CT, antegrade or retrograde pyelography and renal scan in selected cases only. In addition, in patients scheduled for Boari bladder flap, bladder capacity was measured preoperatively with a gravity cystogram and had to be >300 mL to be eligible.

All surgical procedures were performed with either an open or a robot‐assisted approach by a single surgeon (VF), who has extensive experience in both open and robot‐assisted surgery. Surgical techniques and approach were chosen according to multiple factors, such as clinical scenario, ureteral segment involved, surgeon's preference and robotic platform availability. Indeed, during the study period, the robotic platform was available only after 05/2017. Robotic procedures were performed replicating open surgery principles and techniques. Briefly, uretero‐cystoneostomy was performed according to the Lich‐Gregoire technique. When necessary, psoas hitching was performed fixing the bladder corner to the muscle psoas tendon with a 3–0 absorbable suture. Ureteral reimplantation was performed via a direct anastomosis between the most distal vital tract of the ureter and the bladder or Boari flap with 4–0 absorbable sutures. Uretero‐ureterostomy was performed by anastomosing spatulated vital ends of the ureter with 4–0 absorbable sutures. When using the robotic platform, near‐infrared fluorescence imaging with or without concomitant ureteroscopy was used to identify the exact length of the injured ureteral tract. Pyelo‐ureteroplasty was performed according to the Anderson–Hynes technique. Ileal ureteral replacement was performed according to the Yang‐Monti technique. A cystogram was usually performed on postoperative day 5 to 7 depending on the different type of reconstructive procedure performed, and the urethral catheter was removed the following day if no, or only mild, leakage was present.

The following perioperative parameters were collected: type of reconstructive procedure, type of approach, operating room (OR) time, estimated blood loss (EBL), intraoperative complications, urethral catheter indwelling time, upper tract drainage indwelling time and length of stay (LOS).

After discharge, all patients were followed for >6 months. Follow‐up visits were planned 1 week, 1 month and then every 3 months postoperatively. All patients underwent abdominal ultrasound 3 months and abdominal CT 6 months postoperatively. Then, patients were followed with abdominal ultrasound every 6 months and abdominal CT every 12 months. Only patients with high suspicion of significant renal function impairment underwent a renal scan.

Postoperative 90‐day complications were classified according to the modified Clavien system, and further categorised as minor (grades 1 and 2) and major (grades 3 and 4).^19^ Moreover, serum creatinine and eGFR values were assessed at the last follow‐up visit, as well as storage lower urinary tract symptoms (LUTS) with a nonstructured interview in patients who had received a Boari bladder flap. Treatment failure was defined as the occurrence/persistence of upper tract obstruction on abdominal CT due to ureteral stricture requiring a permanent upper tract drainage.

Parametric continuous variables were reported as mean ± standard deviation, whereas median and interquartile range (IQR) were used for nonparametric continuous variables. Frequencies and proportions were reported for categorical variables. The Kruskal–Wallis *H*‐test and the Mann–Whitney *U*‐test were used to compare continuous nonparametric variables, as appropriate. Pearson's chi‐squared test was used to compare categorical variables. Comparison between preoperative and postoperative median values of serum creatinine and eGFR was performed using the Wilcoxon test. We planned a subgroup analysis comparing outcomes between open and robot‐assisted procedures. All clinical records were prospectively collected in a dedicated database. Statistical analyses were performed using the SPSS version 23.0 software (IBM Corp., Armonk, NY, USA). All reported *p*‐values are two‐sided and statistical significance was set at *p* < 0.05.

## 3 RESULTS

During the study period, 59 patients underwent ureteral reconstruction for iatrogenic ureteral injuries. Preoperative characteristics are reported in Table 1. Aetiology was mostly endourological procedures (44%). Reconstruction was immediate and delayed in 10 (16.9%) and 49 (83.1%) patients, respectively. In the latter, a percutaneous nephrostomy tube and a retrograde ureteral stent were placed in 45 (91.8%) and four (8.2%) cases, respectively.

**TABLE 1:** Preoperative characteristics of the 59 patients who underwent ureteral reconstruction for iatrogenic injuries.

Variables	Values
Median (IQR) age (years)	59 (48–70)
Gender, *n* (%)	
Male	31 (52.5%)
Female	28 (47.5%)
Median (IQR) body mass index (kg/m^2^)	25 (22–28)
Age‐adjusted Charlson score, *n* (%)	
0	24 (40.7%)
1	7 (11.9%)
≥2	28 (47.5%)
Aetiology, *n* (%)	
Endourological procedures	26 (44.1%)
Gynaecological procedures	15 (25.4%)
Colonic surgery	4 (6.8%)
Vascular surgery	3 (5.1%)
Urological surgery	10 (16.9%)
Spine surgery	1 (1.7%)
Side, *n* (%)	
Right	26 (44.1%)
Left	31 (52.5%)
Bilateral	2 (3.4%)
Ureteral segment, *n* (%)	
Lumbar	24 (40.7%)
Iliac	8 (13.6%)
Pelvic	27 (45.8%)
Contralateral kidney, *n* (%)	
Normal	55 (93.3%)
Functional impairment	4 (6.8%)
Median (IQR) serum creatinine (mg/dL)	0.9 (0.8–1.1)
Median (IQR) eGFR (mL/min/1.73 m^2^)	82.4 (59.3–101.6)

Abbreviations: eGFR, estimated glomerular filtration rate; IQR, interquartile range.

Uretero‐cystoneostomy was performed in 24 (40.7%), bladder psoas hitch in 5 (8.5%), Boari bladder flap in 19 (32.2%), uretero‐ureterostomy in 6 (10.2%), ileal ureter replacement in 2 (3.4%) and pyelo‐ureteroplasty in 3 (5.1%) patients. The proximal end of Boari bladder flap was at the level of the fifth lumbar vertebra in 6 (31.5%) patients and cranial to it in 13 (68.5%) patients, including two cases with a complete reconstruction after ureteral avulsion. Forty (67.8%) and 19 (32.2%) procedures were performed using an open and robot‐assisted approach, respectively. Details are reported in Table 2.

**TABLE 2:** Ureteral reconstruction procedures in the 59 patients with iatrogenic injuries stratified by aetiology, injury location and surgical approach.

Variables	Uretero‐cystoneostomy	Bladder psoas hitch	Boari bladder flap	Uretero‐ureterostomy	Ileal ureter	Pyelo‐ureteroplasty	*p*‐value
	(*n =* 24)	(*n =* 5)	(*n =* 19)	(*n =* 6)	(*n =* 2)	(*n =* 3)	
Aetiology							<0.001
Endourological procedures (*n =* 26)^a^	9 (37.5%)	2 (40%)	9 (47.4%)	4 (66.7%)	0	2 (66.7%)	
Gynaecological procedures (*n =* 15)	7 (29.5%)	3 (60%)	5 (26.3%)	0	0	0	
Colonic surgery (*n =* 4)	0	0	3 (15.8%)	1 (16.7%)	0	0	
Vascular surgery (*n =* 3)	0	0	2 (10.5%)	1 (16.7%)	0	0	
Urological surgery (*n =* 10)^b^	8 (33.3%)	0	0	0	1 (50%)	1 (33.3%)	
Spine surgery (*n =* 1)	0	0	0	0	1 (50%)	0	
Ureteral segment							<0.001
Lumbar (*n =* 24)	0	0	13 (68.4%)	6 (100%)	2 (100%)	3 (100%)	
Iliac (*n =* 8)	0	2 (40%)	6 (31.6%)	0	0	0	
Pelvic (*n =* 27)	24 (100%)	3 (60%)	0	0	0	0	
Approach							0.82
Open (*n =* 40)	16 (66.7%)	4 (80%)	13 (68.4%)	3 (50%)	2 (100%)	2 (66.7%)	
Robot‐assisted (*n =* 19)	8 (33.3%)	1 (20%)	6 (31.6%)	3 (50%)	0	1 (33.3%)	

^a^ Ureteroscopy (*n =* 24), percutaneous nephrolithotomy (*n =* 2).

^b^ Radical prostatectomy (*n =* 3), simple prostatectomy (*n =* 1), retroperitoneal lymphadenectomy (*n =* 1), renal transplantation (*n =* 1), partial nephrectomy (*n =* 1), previous ureteral reimplantation for bilateral vesico‐ureteral reflux (*n =* 1), transurethral resection of bladder tumour (*n =* 2).

Only one (1.7%) patient experienced an intraoperative complication (bowel lesion during ureteral isolation). OR time and LOS were significantly longer (*p =* 0.004 for both) for more complex surgical procedures, such as Boari bladder flap, uretero‐ureterostomy and ileal ureter replacement, compared with uretero‐cystoneostomy and pyelo‐ureteroplasty. Moreover, surgical techniques requiring bladder opening were associated with a longer urethral catheter indwelling time (*p =* 0.004). No significant differences were observed in EBL, mobilization, return of bowel activity and upper tract drainage indwelling time. Postoperative 90‐day complications occurred in 13 (22%) patients and were minor (grades 1 and 2) in six (10.2%) and major (grades 3 and 4) in seven (11.9%). Major complications were significantly more frequent in patients who underwent Boari bladder flap and uretero‐ureterostomy (*p =* 0.002). Details are reported in Table 3.

**TABLE 3:** Perioperative outcomes, postoperative complications and treatment failure after ureteral reconstruction in the 59 patients with iatrogenic injuries stratified by individual surgical technique.

Variables	Uretero‐cystoneostomy (*n =* 24)	Bladder psoas hitch (*n =* 5)	Boari bladder flap (*n =* 19)	Uretero‐ureterostomy (*n =* 6)	Ileal ureter (*n =* 2)	Pyelo‐ureteroplasty (*n =* 3)	*p*‐value
Median (IQR) operating room time (min)	188 (127–236)	240 (180–291)	260 (240–320)	262 (225–337)	252	200	0.004
Median (IQR) estimated blood loss (mL)	0 (0–100)	0 (0–150)	0 (0–100)	75 (0–325)	225	100	0.22
Median (IQR) time to mobilization (days)	3 (2–4)	4 (2–4)	4 (3–5)	4 (3–5)	5	3	0.03
Median (IQR) time to stool passage (days)	4 (3–6)	5 (3.5–7.5)	5 (3–7)	3 (1.75–4.7)	4	5	0.49
Median (IQR) urethral catheter indwelling time (days)	7 (6–9)	9 (7–11)	10 (8–19)	6 (4–15)	5	7	0.004
Median (IQR) length of stay (days)	9 (8–11)	11 (9–13)	14 (11–22)	9 (6–17)	25	9	0.004
Median (IQR) upper tract drainage indwelling time (days)	35 (9–60)	20 (9–48)	21 (10–80)	60 (55–84)	45	40	0.31
Postoperative complications, *n* (%)							0.002
Minor (grades 1 and 2)	1 (4.2%)	‐	2 (10.5%)	‐	1 (50%)	2 (16.7%)	
Major (grades 3 and 4)^a^	‐		6 (31.6%)	1 (16.7%)	‐	‐	
Treatment failure, *n* (%)							0.17
Permanent ureteral stent	1 (4.2%)	0	2 (10.5%)	3 (50%)	0	0	
Permanent percutaneous nephrostomy tube	1 (4.2%)						

Abbreviation: IQR, interquartile range.

^a^ Grade 3 complications were urinary leakage requiring temporary ureteral stent (*n =* 4) and wound infection and dehiscence requiring surgical revision (*n =* 2); grade 4 complication was myocardial infarction requiring admittance to intensive care unit (*n =* 1).

At a median follow‐up of 42 (IQR 20–62) months, both median serum creatinine level (0.8 [IQR 0.7–1.1] mg/dl) and eGFR (85.6 [IQR 63.4–105] mL/min/1.73 m^2^) were comparable with preoperative values (*p =* 0.44 and 0.17, respectively). Seven (11.9%) patients had a treatment failure. Although higher failure rates were reported in patients who underwent uretero‐ureterostomy (50%) and Boari bladder flap (10.5%), the difference with the other surgical techniques was not statistically significant (*p =* 0.17) (Table 3). Considering Boari bladder flap only, mild storage LUTS were recorded in those six patients with the flap extended at the level of the fifth lumbar vertebra. Conversely, mild to moderate storage LUTS were reported by six out of 13 remaining patients with a more proximally extended flap. In detail, median International Prostate Symptom Score score was 2.5 (IQR 2–3) for the former and 6 (IQR 5–8) for the latter subgroup (*p* < 0.001).

In the comparative analysis between open and robot‐assisted procedures, patients who underwent ileal ureter replacement (*n =* 2) or super‐extended Boari bladder flap for ureteral avulsion (*n =* 2) that were performed with open approach only were excluded. Robot‐assisted procedures were associated with lower EBL (*p =* 0.01), shorter urethral catheter indwelling time (*p =* 0.03) and shorter LOS (p < 0.001) compared with open ones. Treatment failure rate was lower for the former (0/19 vs. 7/36, *p =* 0.04). Details are reported in the supporting information Table S1.

## 4 DISCUSSION

Several findings of our study are noteworthy. First, most iatrogenic ureteral injuries in our series were due to endourological procedures. Interestingly, a recent population‐based study reported the occurrence of ureteral injuries in 2.9% of patients after ureteroscopy, 1.5% after shock wave lithotripsy and 2.6% after shock wave lithotripsy and ureteroscopy.^20^ Of the last patients, 35% required upper tract drainage, 21% endoscopic intervention, 4.8% reconstructive surgery and 1.7% nephrectomy. These data have significant implications as to the importance of a thorough preoperative counselling of patients with upper tract stones scheduled for endourological treatment.

Second, when pooling all procedures, the risk of major complications and treatment failure was low. Obviously, outcomes have to be assessed on a per‐procedure basis, considering that some procedures were complex reconstructive surgical acts. Uretero‐neocystotomy can be performed with or without an antireflux technique.^9^, ^10^ We always perform a direct, refluxing uretero‐neocystotomy and observed two (8.3%) failures. The former occurred in a woman with a pelvic ureteral injury and a concomitant extensive bladder injury following laparoscopic hysterectomy. The latter was reported in a man with distal ureteral stricture after kidney transplantation. In both cases, an impaired blood supply to the distal ureteral portion, rather than our technique, might be considered the cause of failure. In the presence of larger ureteral defects, psoas hitch technique is an essential manoeuvre ensuring a tension‐free uretero‐neocystotomy anastomosis and can be easily performed also using a minimally invasive approach.^10^ Our data confirmed the high success rate of this technique as shown by recent retrospective and prospective surgical series.^9^, ^21^

In cases where psoas hitch is not sufficient for a tension‐free anastomosis, a Boari bladder flap can restore the upper tract continuity, with reported success rate ranging 95% to 100%.^22^ As previously reported,^3^ Boari bladder flap represents our preferred technique to repair ureteral injuries of iliac and lumbar segments. Only two (10.5%) of these patients had a failure. The former occurred in a man treated for recurrent stricture after uretero‐ureterostomy for a lumbar ureteral injury following ureteroscopy. The latter was a woman with a bilateral ureteral injury after open radical hysterectomy for cervical cancer. In this case, we performed a bilateral open Boari flap with a failure observed on the right side. Conversely, neither patient who underwent an open super‐extended Boari bladder flap for ureteral avulsion after ureteroscopy failed. Our results confirmed those reported by Shchukin et al. in a large series of 70 cases treated with open Boari bladder flap for both pelvic and mid‐ureteral strictures.^23^ Recently, several authors reported small series on robot‐assisted ureteral reimplantation using Boari bladder flap.^24^ In 2016, Stolzenburg reported on 11 consecutive patients who underwent robot‐assisted Boari flap with durable repair of their distal strictures at 15‐month follow‐up.^16^ More recently, Dell'Oglio et al. reported no failure in seven patients who underwent robotic Boari flap for ureteral strictures of different aetiology at a minimum follow‐up of 1 year.^9^ In our series, no failure was observed for robot‐assisted Boari bladder flap at a median follow‐up of 11 months.

Uretero‐ureterostomy is mainly used to repair short defects involving the mid‐ureter. Although historical open series reported a very high success rate,^22^ a recent publication identified uretero‐ureterostomy as an independent predictor of failure after reconstructive surgery for any‐cause injuries.^25^ In our series, two open cases failed and two robotic did not. Preservation of good vascular supply to ureteral ends and creation of a wide, tension‐free anastomosis are the key to success. Promising results have been reported for robot‐assisted uretero‐ureterostomy with a patency rate of up to 100%.^2^, ^26^ We speculate that intraoperative near‐infrared fluorescence may help identify the exact length of the damaged ureter and check the vascularization status of both ureteral ends.

Other options to bridge long ureteral defects include the use of ileum, appendix or colon as ureteral replacement. In our series, only two patients required an (open) ileal ureteral replacement, with no failure. Potential bowel‐related complications should be seriously considered, and these techniques should be discussed only in highly complex cases where other options are not applicable.

Third, in our series, a robot‐assisted approach was used in approximately one out of three patients. Uretero‐cystoneostomy with or without bladder psoas hitch and Boari bladder flap were the most frequently performed robotic procedures. The robot‐assisted approach was associated with significantly lower EBL, shorter urethral catheter indwelling time and LOS and lower failure rate. Our data confirmed those of a meta‐analysis of three studies comparing open and robot‐assisted ureteral reconstructions, which showed better perioperative outcomes for the robotic approach.^24^ Possible explanations for this finding are enhanced precision and near‐infrared fluorescence imaging capabilities of robotic platforms, which are of value in conditions where very often anatomy is distorted and blood supply is compromised. However, the detected difference in catheter indwelling time between might rather depend on the different types of reconstructive procedure performed, also considering that in the open surgery subgroup, there was a higher number of cases with extended Boari flap potentially requiring a longer indwelling time. Currently, we favour a robotic approach in patients with iatrogenic ureteral injuries secondary to endourological or laparoscopic procedures and an open approach after initial open surgery.

Our study has the limitations inherent to the retrospective analysis of single‐centre, single‐surgeon cohort. Moreover, the adopted surgical procedures were heterogeneous according to the clinical scenarios encountered and surgeon's preferences. Finally, the comparative analyses between the different surgical techniques and between open and robot‐assisted approach are clearly underpowered and limited by the small number of cases included.

In conclusion, our study showed that endourological procedures represent an increasing cause of severe iatrogenic ureteral injuries requiring surgical reconstruction. The choice of the most appropriate procedure depends on ureteral injury aetiology, location and extent, as well as surgeon's preference. Robot‐assisted ureteral reconstruction should be preferred given the associated better perioperative outcomes and lower failure rates. Larger multicentre, multisurgeon, ideally prospective studies are required to improve our knowledge on this rare condition with potentially devastating sequelae and relevant medico‐legal ramifications. Until then, our study may serve as a pragmatic guide to decision‐making for patients with iatrogenic ureteral injuries considering reconstruction.

## AUTHOR CONTRIBUTIONS

**Marta Rossanese:** Data curation and writing original draft. **Gianluca Giannarini:** Writing review and editing and investigation. **Riccardo Scalia:** Data curation. **Luciano Macchione:** Data curation. **Alessandro Crestani:** Data curation and investigation. **Alchiede Simonato:** Supervision. **Vincenzo Ficarra:** Conceptualization; Formal analysis and supervision.

## CONFLICT OF INTEREST STATEMENT

All authors declare no conflicts of interests.

## Supporting information


**Table S1.** Ureteral reconstructive procedures and perioperative outcomes in 55 patients with iatrogenic injuries stratified by surgical approach.*
