# Supplementary material for: Outcomes and treatment failure after open or robotic ureteral reconstruction for iatrogenic injuries
Source: BJUI Compass. 2023 Jul 4;4(6):673–9. doi: 10.1002/bco2.267 (PMC10560615; doi:10.1002/bco2.267)
Supplement: Supplementary file 1 — Table S1. Ureteral reconstructive procedures and perioperative outcomes in 55 patients with iatrogenic injuries stratified by surgical approach.* [file BCO2-4-673-s001.docx]

Supplementary Table 1. Ureteral reconstructive procedures and perioperative outcomes in 55 patients with iatrogenic injuries stratified by surgical approach.*

| Variables | Open  approach  (n = 36) | Robot-assisted approach  (n = 19) | p value |
| --- | --- | --- | --- |
| Surgical technique, n (%)  - uretero-cystoneostomy  - uretero-cystoneostomy with bladder psoas hitch  - Boari bladder flap  - uretero-ureterostomy  - pyelo-ureterostomy | 16 (44.4%)  4 (11.1%)  11 (30.6%)  3 (8.3%)  2 (5.6%) | 8 (42.1%)  1 (5.3%)  6 (31.6%)  3 (15.8%)  1 (5.3%) | 0.89 |
| Median (IQR) operating room time (min) | 226  (146 – 260) | 245  (215 – 270) | 0.07 |
| Median (IQR) estimated blood loss (ml) | 75  (0 – 145) | 0  (0 – 50) | 0.01 |
| Median (IQR) time to mobilization (days) | 4  (3 – 4) | 3  (3 – 4) | 0.37 |
| Median (IQR) time to stool passage (days) | 4.5  (3 – 7) | 4  (3 – 5) | 0.10 |
| Median (IQR) urethral catheter indwelling time (days) | 8.5  (7 – 13) | 7  (6 – 9) | 0.03 |
| Median (IQR) length of stay (days) | 12  (9 – 14) | 8  (8 – 10) | <0.001 |
| Postoperative complication, n (%)  - minor (grade 1-2)  - major (grade 3-4) | 3 (8.3%)  4 (11.1%) | 2 (10.5%)  1 (5.3%) | 0.73 |
| Treatment failure, n (%) | 7 (19.4%) | 0 | 0.04 |

IQR = interquartile range

* of the 59 total patients, those who underwent ileal ureter replacement (n=2) or super-extended Boari bladder flap (n=2) that were performed with open approach only, were excluded
